# Draft Genome Sequence of Polychlorinated Biphenyl-Dechlorinating *Dehalococcoides mccartyi* Strain SG1, Which Carries a Circular Putative Plasmid

**DOI:** 10.1128/genomeA.00901-14

**Published:** 2014-10-02

**Authors:** Shanquan Wang, Kern Rei Chng, Chen Wu, Andreas Wilm, Niranjan Nagarajan, Jianzhong He

**Affiliations:** a Department of Civil and Environmental Engineering, National University of Singapore, Singapore; b Computational and Systems Biology, Genome Institute of Singapore, Singapore

## Abstract

*Dehalococcoides mccartyi* strain SG1, isolated from digester sludge, dechlorinates polychlorinated biphenyls (PCBs) to lower congeners. Here we report the draft genome sequence of SG1, which carries a 22.65 kbp circular putative plasmid.

## GENOME ANNOUNCEMENT

Persistent and toxic polychlorinated biphenyls (PCBs) were massively produced as commercial mixtures for industrial uses, leading to the contamination of sediments of rivers, lakes, and harbors worldwide. The obligate organohalide-respiring *Dehalococcoides mccartyi* strains in both microcosms (1–4) and pure cultures (5–7) have been identified to be capable of dechlorinating PCBs in distinct patterns. Populations of this bacterial group have small circular chromosomes of around 1.5 Mbp, yet each carries a suite of 10 to 36 different *rdhA* genes encoding reductive dehalogenases (RDases) for catalyzing the chlorine removal (8). Most RDase genes are located in the high plasticity regions (HPRs) of chromosomes (7, 9, 10), suggesting that their horizontal acquisition is possibly through temperate bacteriophages or plasmids. To date, however, no plasmid has been found in *Dehalococcoides*.

Strain SG1 belonging to the *Dehalococcoides* Pinellas subgroup was isolated from a PCB-dechlorinating sediment-free culture originated from digester sludge of an industrial wastewater treatment plant in Singapore (2). The genome of strain SG1 was sequenced by using Hiseq 2000 from pair-end libraries with an average insert size of 300 bp and a read length of 76 bp. The reads were assembled using SOAPdenovo (11) and scaffolding was done with Opera (12). The coverage for the genome sequence assembly was 1,580×. Gapcloser (11) was utilized for *in silico* closing of gaps between contigs, which generated 5 scaffolds. Finally, targeted PCR reactions and Sanger sequencing were used to confirm an independent circular piece of DNA, possibly a putative plasmid. Open reading frames (ORFs) were predicted using Prodigal (13). Functional annotations were assigned by screening predicted ORFs with entries in the KEGG database (14) using RapSearch (15).

The assembled draft genome of strain SG1 is 1,428,734 bp long, with a G+C content of 47.05%. It contains 1,486 protein-coding genes (including 28 RDase genes), which are similar to those of other sequenced *Dehalococcoides mccartyi* strains (7, 9, 10). The significant difference is that SG1 carries a 22.65 kbp circular putative plasmid. This putative plasmid is predicted to encode 26 proteins, including integrase, metallophosphoesterase, DNA primase, and a transcriptional regulator. Nine of the 26 protein coding sequences share 61% to 96% similarities with their homologues in the chromosome of *Dehalococcoides mccartyi* 195. The genome sequence of strain SG1 may provide new insights into the adaption mechanisms of *Dehalococcoides* to the organohalide respiration of PCBs.

### Nucleotide sequence accession numbers.

This whole-genome shotgun project has been deposited at DDBJ/EMBL/GenBank under accession no. JPRE00000000. The version described in this paper is version JPRE01000000.
